# Toxocariasis in Cuba: A Literature Review

**DOI:** 10.1371/journal.pntd.0001382

**Published:** 2012-02-28

**Authors:** Idalia Sariego, Kirezi Kanobana, Lázara Rojas, Niko Speybroeck, Katja Polman, Fidel A. Núñez

**Affiliations:** 1 Institute of Tropical Medicine “Pedro Kourí”, Havana, Cuba; 2 Institute of Tropical Medicine, Antwerp, Belgium; 3 Institute of Health and Society, Université Catholique de Louvain, Brussels, Belgium; University of Oklahoma Health Sciences Center, United States of America

## Abstract

Human toxocariasis (HT) is a zoonotic disease caused by infection with the larval stage of *Toxocara canis*, the intestinal roundworm of dogs. Infection can be associated with a wide clinical spectrum varying from asymptomatic to severe organ injury. While the incidence of symptomatic human toxocariasis appears to be low, infection of the human population is widespread. In Cuba, a clear overview on the status of the disease is lacking. Here, we review the available information on toxocariasis in Cuba as a first step to estimate the importance of the disease in the country. Findings are discussed and put in a broader perspective. Data gaps are identified and suggestions on how to address these are presented. The available country data suggest that *Toxocara* infection of the definitive dog host and environmental contamination with *Toxocara* spp. eggs is substantial, but information on HT is less conclusive. The availability of adequate diagnostic tools in the country should be guaranteed. Dedicated studies are needed for a reliable assessment of the impact of toxocariasis in Cuba and the design of prevention or control strategies.

## Introduction

Human toxocariasis (HT) is one of the most common human parasitic infections in the world, affecting mainly the poorest communities of developing countries. It is caused by zoonotic infection with the larval stage of *Toxocara canis*, the intestinal roundworms of dogs, and probably by the roundworms of cats (*Toxocara cati*) as well [1]. Although the disease can be significant and debilitating, the incidence of severe clinical manifestations is unknown, and diagnosis is difficult. This leads to a false perception that the burden and public health impact are low and consequently results in the classification of HT as a neglected zoonosis.

HT is acquired by the ingestion of eggs, which originate from the feces of the definite dog host and embryonate in the environment [2] (Figure S1). Children in their first decade of life are prone to infection because of their geophagic behavior and mouthing of objects, which is linked to a higher risk of exposure at playgrounds or sandboxes contaminated with dog feces [3]. HT is mostly asymptomatic but can be associated with severe clinical syndromes due to organ injury by migrating larvae [4]. Depending on the organs affected and the specificity of the symptoms, the predominant clinical syndromes are classified as visceral larva migrans (VLM), ocular larva migrans (OLM), and common, neurologic, and covert toxocariasis [5]. Diagnosis of HT is traditionally based on a combination of clinical and histopathological interpretations. Yet, sensitivity is low, as biopsy material may not always contain the larvae. Serology, using in vitro–obtained excretory-secretory products of the larvae (TES), is the best laboratory-based option for diagnosis [6], and is considered a useful predictor of *T. canis* infection when coupled to relevant clinical data. Worldwide, reported *Toxocara* seroprevalence data among apparently healthy individuals range (using either ELISA or western blot) from 2.4% in Denmark [7] to 92.8% in La Réunion [8].

In Cuba, there are an estimated 2 million dogs that have access to veterinary control. However, only 40% of these participate in the rabies vaccination program, illustrating the low compliance to recommended veterinary prevention [9]. Consequently, a large proportion of dogs are born with congenital toxocariasis. Combined with the high numbers of stray dogs that are not routinely dewormed, this points to a rich potential for environmental contamination and subsequent human exposure. However, a clear idea of the status and importance of toxocariasis within the country is lacking. To this end, we recently started a project dedicated to the epidemiology and the diagnosis of toxocariasis in Cuba. As a first step, we reviewed the available information on *Toxocara* in Cuba and put it in a broader perspective, identifying data gaps that should be addressed.

## Methods

**Figure 1:**
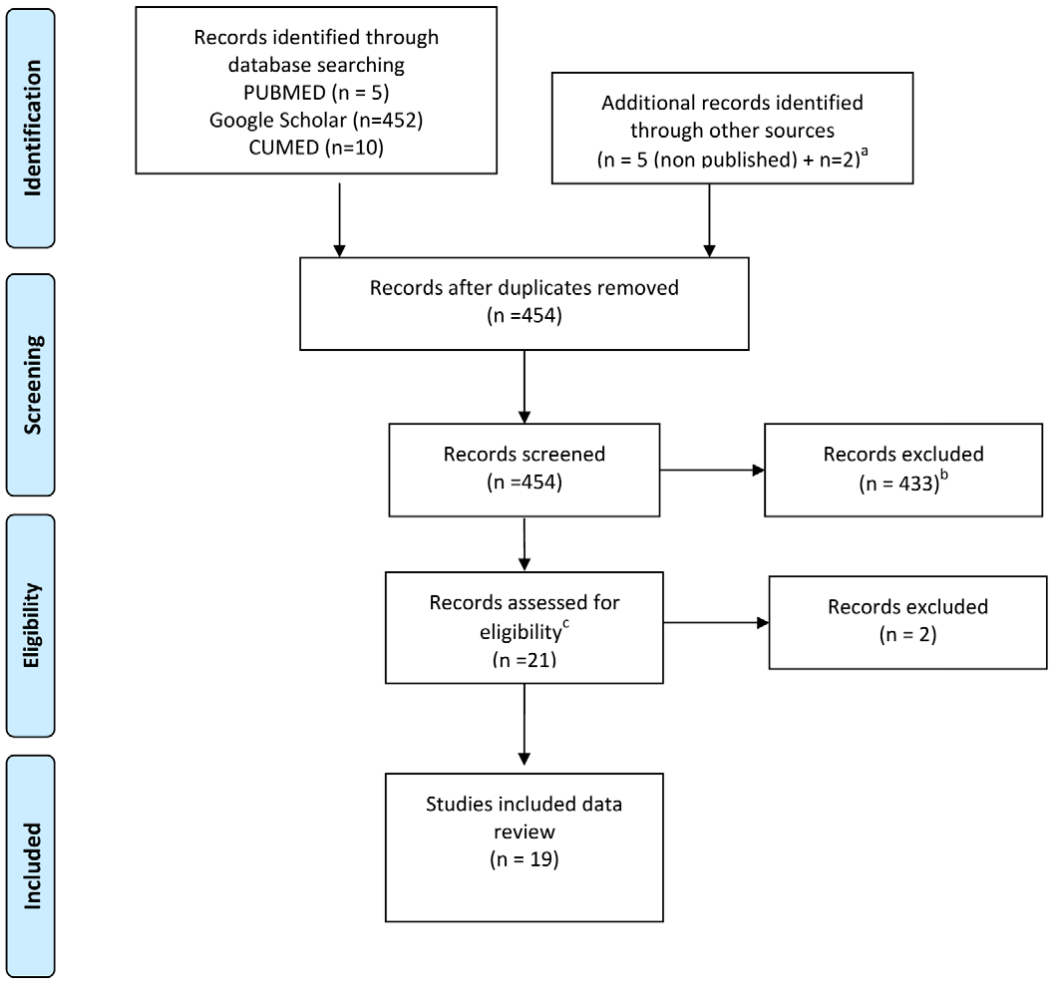
Flow diagram of literature searches. ^a^Additional records consisted of five yearly reports on *Toxocara* antibody detection in patients suspected of VLM or OLM; one book chapter on VLM [33] and a master's thesis on toxocariasis in dogs [11]. ^b^Reasons for exclusion were: 1. non-relevant association between the keywords (60%) (e.g., Cuba as name of the author, reports on *Toxocara* published in a Cuban journal, paper, or reference to paper conducted in Cuba in which toxocariasis is mentioned as differential diagnosis, papers on *Toxocara vitolorum*, etc.); 2. reference to a Cuban report on *Toxocara* seroprevalence data of Cuba (20%); and 3. replicates of the same report within the Google Scholar search (16%). One record was excluded because none of the co-authors was familiar with the language. ^c^Eligibility criteria were: 1. subject toxocara, toxocariasis, or larva migrans irrespective of the field or type of publication; and 2. new data about Cuba.

PubMed, Google Scholar, ISI Web of Knowledge, and CUMED databases were searched using combinations of keywords “toxocara”, “toxocariasis”, “larva migrans”, and “Cuba”. The search was conducted in February 2010 and was limited neither by language, study design, nor date of publication. The CUMED database contains all Cuban scientific publications in human medicine and related fields. Non-peer-reviewed articles and theses, books, etc. were searched in local libraries and websites. Bibliographies were screened and researchers were contacted to provide information and/or data not included in published records. Figure 1 shows the flow diagram of the literature search. The ISI Web of Knowledge did not yield additional relevant records and results are not included in the diagram. Published articles and non-published data or documents were not excluded with regard to potential biases or study design, as the goal was to retrieve as much information as possible. After clearance of irrelevant documents, 19 reports (including both published and non-published data) were considered eligible to be included in the present review.

## *T. canis* Infections in the Final Dog Host

We identified three reports: two papers published in peer-reviewed journals, and one master's thesis (Table 1). The first Cuban study on *T. canis* infection in dogs originates from 1992 and was a cross-sectional study conducted to determine the prevalence in owned dogs of Havana. Feces were sampled from 22 randomly selected dogs in each of the 15 municipalities of Havana [10]. Eggs in the dogs' feces were detected by direct microscopy and the Willis brine flotation method. The overall prevalence of infected dogs was 17.9% (95% CI: 13.9–22.4, *n* = 59 out of 330), but figures ranged from 4.5% (95% CI: 0.1–22.8, *n* = 1 out of 22) to 40.9% (95% CI: 20.7–63.6, *n* = 9 out of 22) across the municipalities, with higher prevalences in dogs originating from highly populated parts of the city.

**Table 1:** Chronological overview of reports on *T. canis* infection in dogs in Cuba.

Date (Location)	Author	Type of Publication	Summary	Ref.
1992 (Havana city)	Duménigo B, et al.	Peer review	Twenty-two randomly selected owned dogs were sampled in each municipality of Havana. Two grams of feces from each dog were analyzed by direct microscopy examination and Willis brine flotation method. Fifty-nine dogs out of 330 (17.9%) were infected with *T. canis*.	[10]
2003 (Villa Clara province)	De la Fe PY	Master's thesis	Using a random sampling method across all municipalities of Villa Clara province, *Toxocara* infection was investigated by coprology in 321 owned dogs. An apparent prevalence of 27.1% was measured.	[11]
2005–2006 (Havana city)	Hernández R, et al.	Peer review	Four hundred sixty-one stray dogs from Havana were included in the study. Upon euthanasia, the intestine was dissected and examined for the presence of helminths. The frequency of infection with *T. canis* was 19.7%.	[9]

A second study was part of a 2004 master's thesis of a veterinarian postgraduate student at the Institute of Tropical Medicine “Pedro Kourí” (IPK) in Havana [11]. This study was conducted in the province of Villa Clara, located in the central region of the island. Based on the total number of dogs in the province, provided by the provincial Institute of Veterinary Medicine, a total sample size of 321 dogs was assessed. Subsequently, a random sample of dogs was selected per municipality, proportional to the total number of owned dogs in that municipality. Feces were sampled and coprological examination was conducted. Parasite eggs of *T. canis* were detected in 27.1% of the animals (95% CI: 22.3–32.3, *n* = 87 out of 321). The proportion of infected dogs was significantly higher in dogs below 1 year of age (62.1% [95% CI: 52.6–70.9, *n* = 72 out of 116]) as compared to older dogs (7.3% [95% CI: 4.1–11.8, *n* = 15 out of 205, *p*≤0.05]).

A third and more recent study carried out in 2005 focused on the detection of zoonotic helminths in stray dogs of Havana in two different periods of the year [9]. In this study, the prevalence of *T. canis* was determined by the detection of adult worms in the intestine of dogs, and not by the detection of eggs in feces. Adult *T. canis* worms were detected in 19.7% of the dogs investigated (95% CI: 16.2–23.7, *n* = 91 out of 461). In accordance with the above study, the prevalence was higher in animals younger than 1 year (25.4% versus 13.15% for dogs below or above 1 year of age, respectively; OR 2.25 [95% CI: 1.37–3.67; *p*≤0.01]). Moreover, the prevalence of the parasite in females was significantly higher as compared to male dogs (28.8% [95% CI: 22.5–34.0] versus 9.9% [95% CI: 6.3–14.8, *p*≤0.05] for females and male dogs, respectively). The authors observed no differences in apparent prevalences of *T. canis* infection in dogs between the rainy (May–October) and the dry (November–April) season.

These data suggest that up to one-fifth of the dogs in Cuba could be infected with *T. canis*. Despite a more than 10-year interval, the two studies accomplished in Havana city yielded similar prevalence estimates, indicating that the situation has not changed over the years. Comparable prevalences in Villa Clara and Havana point to presumably small differences between regions in the country. Reported point prevalences of infection in dogs range from 0.7% to 82.6% worldwide, depending both on the methods used and on the location (reviewed by [12]). The estimates for Cuba approximate figures measured in other Caribbean countries. Prevalences of 25% and 32% were reported in dogs from Oranjestead, Aruba, and Anse-la-Raye, St. Lucia, respectively [13].

The best option for reducing infection levels in dogs is preventive chemotherapy [14]. To be effective, this intervention should reach both owned and stray dogs. Control programs for stray dogs are in place in Cuba, but they do not reach the full dog population (referred to by [9]). Yet, the presence of potentially infected dogs remains the main determinant for environmental contamination with *Toxocara* spp. eggs [3].

## Environmental Contamination with *Toxocara* spp. Eggs

We identified two reports conducted in Havana in the mid-1990s and one chapter of a master thesis on a study conducted in 2002 in Villa Clara (Table 2). The first Cuban study dealing with environmental contamination detected *Toxocara* spp. eggs in 19 out of 45 soil samples collected in parks or public areas from residential areas across the 15 municipalities of Havana (three samples each), yielding an overall prevalence of 42.2% infected areas (95% CI: 29.8–62.3) [15]. In 12 municipalities, at least one public area or park was contaminated, resulting in 80% prevalence at the municipality level. Moreover, almost 40% of the eggs recovered were in the embryonated phase.

**Table 2:** Chronological overview of reports on soil contamination with *Toxocara* spp. eggs in Cuba.

Date (Location)	Author	Type of Publication	Summary	Ref.
1995 (Havana city)	Duménigo B and Galvez D	Peer review	Three samples of 20 grams of soil, at a depth of 5 to 10 cm, were taken randomly in each municipality of Havana. Analysis was performed with the conical cup technique and the Willis brine flotation method. Out of 15 municipalities, 12 (80%) were contaminated.	[15]
1995 (Havana city)	Laird RM, et al.	Peer review	A cross-sectional sampling of 218 soil samples of 50 grams each, at a depth of 3 cm, was conducted across the municipalities of Havana. Samples were analyzed using a simple flotation technique. All Havana municipalities were polluted.	[16]
2003 (Villa Clara province)	De la Fe PY	Master's thesis	Two groups of 30 randomly selected houses located within the Villa Clara province were formed. The first group included owned dogs infected with *T. canis*, and the second group, owned non-infected dogs. In each backyard, five soil samples were taken at a depth of 5 to 10 cm. Sheather modified methodology was used for analyzing the samples. The probability of detecting *Toxocara* spp. eggs in backyards of houses with infected dogs (13, 43%) was higher as compared to backyards of houses with non-infected dogs (3, 10%) (*p*≤0.05).	[11]

The second study comprised a cross-sectional sampling of 216 parks and two public areas, located randomly across Havana, but similar to the first study, included all 15 municipalities [16]. Sixty-eight percent of the parks (95% CI: 61.3–74.2) and both public areas were contaminated with *Toxocara* spp. eggs, yielding an overall prevalence of almost 70%. All municipalities were contaminated, but the highest level of contamination was found in the center of and in the older parts of the city. Overall, more than 80% of the eggs recovered were embryonated. Both studies evidence the high level of contamination of parks and public areas and the high proportion of embryonated (and thus infective) eggs. Differences in results are probably due to the higher number of places screened in combination with a more extensive sampling methodology in the study conducted by Laird et al. [16]. Although seasonal sampling would have been more representative [17], the data clearly demonstrate the risk of individuals of being exposed to *Toxocara* spp. eggs.

Finally, the contamination level of backyard samples was assessed in the Villa Clara study [11]. Two groups of 30 randomly selected houses either owning infected or non-infected dogs were formed. In each of the houses, five samples of soil were taken between 5 and 10 cm deep at intervals of 30 cm. Embryonated *Toxocara* spp. eggs were present in 43% and 10% of households with *Toxocara*-infected and non-infected dogs, respectively (*p*≤0.05), showing that the risk of contamination of backyards is higher when, but not restricted to, having an infected dog. In tropical settings, owned dogs often have a free-roaming behavior similar to that of stray dogs [18] and backyards tend to be accessible, which may partly explain the latter results.

## Human Toxocariasis

Eight publications dealing with HT in Cuba were identified (Table 3). The first report on VLM in Cuban patients dates from 1968: at the International Congress of Gastroenterology of Prague, the Cuban gastroenterologist Prof. Llanio presented cases of VLM in adults, which were diagnosed by laparoscopy and subsequently confirmed by hepatic biopsies (referred to by [19]). The next two case reports describe cases of VLM similarly diagnosed by combining laparoscopy and hepatic biopsy (one and seven case reports in 1969 and 1974, respectively) [19], [20]. A review of the clinical history of 15 VLM patients was published 4 years thereafter [21]. In all patients, the diagnosis based on clinical signs and laparoscopy was not conclusive.

**Table 3:** Chronological overview of reports on human toxocariasis in Cuba.

Date (Location)	Author	Type of Publication	Clinical Aspect	Summary	Ref.
1969 (Cuba)	Rodríguez A and Zamora F	Peer review	VLM	The clinical history of a 44-year-old patient with VLM is presented. Diagnosis was conducted by hepatic biopsy following laparoscopy.	[20]
1970–1973 (Cuba)	Fernández JE, et al.	Peer review	VLM	One hundred and five hepatic biopsies performed in the “Dr Carlos J. Finlay” military hospital of Havana are reviewed, of which seven cases of VLM are presented.	[19]
1975–1978 (Cuba)	Pena A, et al.	Peer review	VLM	Fifteen clinical histories of patients with a diagnosis of VLM in “General Calixto García” Hospital of Havana are presented. Most patients were men living in an urban environment. Diagnosis was established by laparoscopy.	[21]
1983 (Cienfuegos province)	Rey S and Sol G	Peer review	VLM	One hundred and thirteen patients with VLM diagnosed by laparoscopy or biliary drainage were examined by ultrasonography in “Dr. Gustavo Aldereguía Lima” hospital in Cienfuegos. One-third of the cases were infected with *F. hepatica*. The authors concluded that ultrasonography contributes to the diagnosis of VLM.	[22]
1988–1990 (Havana)	Montalvo AM, et al.	Peer review	Serological survey	One hundred and fifty-six healthy children (between 1 and 14 years old) of one municipality of Havana were included in a serological survey on toxocariasis. Antibodies to TES antigens were detected in 5.1% of the children.	[27]
2002 (Cuba)	Luis MC, et al.	Peer review	VLM	The clinical history of an 8-year-old boy with glandular larva migrans, is presented. Diagnosis was made upon biopsy of a cervical lymph node.	[23]
2008 (Cienfuegos province)	Curbelo MJ, et al.	Peer review	OLM	A clinical case of OLM is presented. Acute uveitis and a peripheral granuloma in the left eye were diagnosed in a 4-year-old boy. Epidemiologic evidence related to exposure to *Toxocara* was recorded during the anamnesis.	[25]
2009 (Ciego de Avila province)	Delgado M, et al.	Peer review	VLM	Two cases of VLM in children below 6 years of age from the Ciego de Avila province are presented. Both children tested positive in a TES-based ELISA for toxocariasis.	[24]

A publication in the 1980s documented 113 VLM cases diagnosed within a period of 6 months. The outbreak was attributed to the consumption of contaminated vegetables. Approximately one-third of the cases appeared to be infected with *Fasciola hepatica*. Again, the definite cause of the outbreak was not conclusive, as the main emphasis of the study was on assessing the added value of ultrasound in the diagnosis of VLM [22].

Following a gap of more than 15 years, one case report of glandular LM in an 8-year-old boy suffering from clinical manifestations suggestive of toxocaral VLM was published [23]. The presence of a nematode larva was confirmed by biopsy.

The most recent report on VLM dates from 2009 and describes two suspected clinical cases [24]. For the first time, serology (detection of antibodies) was used to support the clinical diagnosis. Both children tested positive in a TES-based IgG ELISA for toxocariasis (Diagnostic Automation, Inc., Calabasas, CA).

The only case report on OLM was published in 2008 and describes the clinical history of a 4-year-old boy presenting with pain and a red eye [25]. The acute uveitis was accompanied by a granuloma in the eye fundus, and successfully treated with a combination of anthelminthics and corticosteroids.

Clinical case reports on HT are relatively well represented, but a definitive diagnosis of toxocariasis is often lacking. The high number of case reports may be caused by the methodology used in our literature search, i.e., inclusion of all case reports on VLM, irrespective of a confirmed etiology. VLM syndrome is a systemic disease that can be attributed to other parasite species besides *Toxocara* alone, e.g., *F. hepatica* [26].

The single epidemiological study on HT in Cuba is a serological survey conducted in a group of healthy children from the municipality of La Lisa in Havana, reporting an apparent prevalence of *T. canis* antibodies of 5.1% (95% CI: 2.2–9.8, *n* = 8 out of 156) [27] using an in-house ELISA technique that, at that time, had been developed in the laboratories of the IPK and that was discontinued some years later. The reported 5.1% seropositivity rate is lower than could have been anticipated regarding the levels of environmental contamination measured in the same area, and in the same period (see above). It is also lower compared to seropositivity rates reported in children of neighboring Caribbean countries with similar climatic conditions: 8.3% found in Puerto Rico [28], 60% in a community of St. Lucia [29], and more recently, 60.3% among schoolchildren of Trinidad and Tobago [30].

Although environmental contamination and *Toxocara* seropositivity rates in humans are intimately related [31], societal factors and risk behaviors of individuals can substantially influence this relationship. Factors such as low socioeconomic status, lack of adequate water supplies, age, and pica are known to increase the risk of humans to ingest eggs that are dwelling in the environment [3], and all these factors should be considered when interpreting data. Unfortunately, this type of information was not available for the latter study, preventing such an analysis.

**Figure 2:**
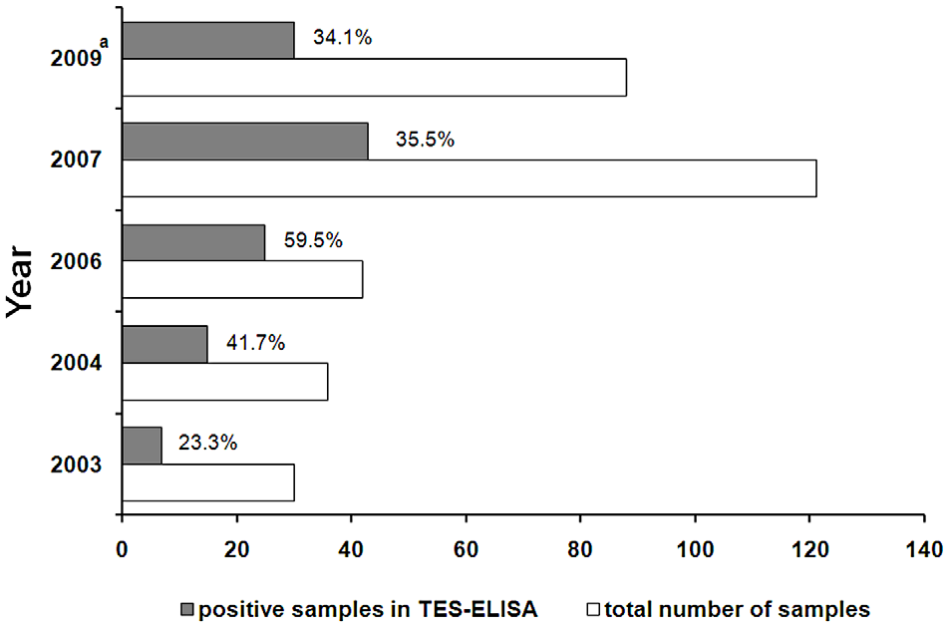
Yearly internal records of the IPK on the serodiagnosis of individuals suspected of human toxocariasis. Proportion of positive samples in a commercial TES-based ELISA (Diagnostic Automation, Inc., Calabasas, CA) performed by the Department of Parasitology of the IPK. Samples were from patients suspected of OLM or VLM and sent by clinicians across the country for serological confirmation of toxocariasis. No data are available for 2005 and 2008 due to inaccessibility of the commercial ELISA because of the trade embargo with the country. Serum samples were tested anonymously, and information on origin or on the differentiation between suspicion of OLM or VLM syndromes is not available, except for 2003 where all samples analyzed originated from the Ophthalmologic Institute “Ramón Pando Ferrer” and thus were suspicious of OLM. Follow-up of patients was not conducted by the IPK, preventing the confirmation of the suspected diagnosis. ^a^Data from 2009 are limited to samples received up to the beginning of August 2009.

Finally, a large set of (indirect) evidence on HT was obtained from yearly reports on serological diagnosis performed at the Department of Parasitology of the IPK, which is the reference lab for diagnosis of toxocariasis within Cuba. Samples from patients suspected of HT are sent by clinicians from all over the country for serological confirmation. This data set has several limitations. Samples originate from suspicious patients and hence do not represent the general Cuban population. Second, the availability of serological tests at the IPK was not always guaranteed because of the trade embargo with the country. This implies that periodically data may be missing. Nevertheless, evaluation of the available data shows antibody prevalences between 23% and 59% (Figure 2). Moreover, the number of samples received for testing has been increasing over the years, which is in line with increased awareness or re-emergence of the parasite within Cuba.

Overall, the data emphasize the major need for reliable, robust, and readily available sero-diagnostic tests for toxocariasis in the country, a prerequisite to obtain a representative estimate of both clinical cases and of background exposure to the parasite.

## Knowledge/Awareness about Toxocariasis in Cuba

We found one preliminary single report on the assessment of the knowledge of toxocariasis among health workers in Cuba: a cross-sectional study carried out in 2005 in Cienfuegos, a city located more in the center of the island [32]. The survey included 51 medical doctors among a total of 108 clinicians, general practitioners, ophthalmologists, parasitologists, and pediatricians of the city. The study design is partially biased because the authors decided to include all ophthalmologists and parasitologists in the sub-sample, specialists who, in their opinion, should have a more extended knowledge of toxocariasis. A questionnaire was submitted to the physicians addressing their knowledge on transmission (five questions), clinical manifestations (two questions), and diagnosis, treatment, and prevention (one question each) of HT. All questions were multiple choice and the maximum value assigned to each question was ten points. Results were considered satisfactory when a score above eight was obtained. Based hereon, the authors concluded that only two aspects of the disease appeared to be sufficiently known, i.e., the clinical picture of HT (55.9% of the physicians) and the treatment of HT (92.2% of the physicians). Less than one-fifth of the physicians knew how the disease could be diagnosed.

The value of this information is limited by the small study sample and the single report. Nevertheless, it shows that in this specific target group (physicians who are either directly or indirectly involved with toxocariasis), knowledge on toxocariasis was limited. This information gap is likely to be even bigger in the general population, emphasizing the need for education on the knowledge and awareness of toxocariasis.

## Conclusion

The literature search on *T. canis* and toxocariasis in Cuba yielded only a limited number of reports. Data issue from case reports, or from small studies, were predominantly conducted in Havana city, hampering a reliable estimation of the importance of toxocariasis in Cuba. Still, the data suggest that *Toxocara* infection of the definitive dog host and environmental contamination with *Toxocara* spp. eggs is substantial. Information on HT is less conclusive. Overall, information is limited and scattered across different fields and over time. There is a clear need for more recent data. The availability of adequate diagnostic tools in the country should be guaranteed. Dedicated studies including veterinary, human, and environmental health data should be conducted, as these are all intimately linked in the epidemiology and control of toxocariasis. The outcome of such studies will allow policy makers to set priorities and design strategies, combining accurate surveillance with prevention rather than cure. With this review we aim to contribute to the advocacy of toxocariasis in Cuba.

**Key Learning Points**

- *Toxocara* infection of the definitive canine dog host and environmental contamination with *Toxocara* spp. eggs are frequent in Cuba.
- Information on human toxocariasis in Cuba is mainly based on clinical case reports; knowledge on the disease is limited.
- A clear picture on the status and public health impact of toxocariasis within Cuba is lacking. Data gaps are caused by limited information that is scattered across different fields and over time.
- Dedicated epidemiological studies, including veterinary, human, and environmental health data are required to improve the understanding of toxocariasis transmission within the Cuban context and develop adequate prevention and control strategies.

**Five Key Papers in the Field**

1. Rubinsky-Elefant G, Hirata CE, Yamamoto JH, Ferreira MU (2010) Human toxocariasis: diagnosis, worldwide seroprevalences and clinical expression of the systemic and ocular forms. Ann Trop Med Parasitol 104: 3–23.
2. Smith H, Holland C, Taylor M, Magnaval JF, Schantz P, Maizels R (2009) How common is human toxocariasis? Towards standardizing our knowledge. Trends Parasitol 25: 182–188.
3. Hernández Merlo R, Núñez FA, Pelayo Durán L (2007) Potencial zoonótico de las infecciones por helmintos intestinales en perros callejeros de Ciudad de La Habana. Rev Cubana Med Trop 59: 234–240.
4. Despommier D (2003) Toxocariasis: clinical aspects, epidemiology, medical ecology, and molecular aspects. Clin Microbiol Rev 16: 265–272.
5. Laird RM, Carballo D, Reyes EM, García T, Prieto V (1995) *Toxocara* sp. en parques y zonas públicas de Ciudad de La Habana. Rev Cubana Hig Epidemiol 58: 116–118.

## Supporting Information

Figure S1 **Life cycle of** ***Toxocara canis***
(JPG)
