# Supplementary figures and images for: Toxocariasis in Cuba: A Literature Review
Source: PLoS Negl Trop Dis. 2012 Feb 28;6(2):e1382. doi: 10.1371/journal.pntd.0001382 (PMC3289590; doi:10.1371/journal.pntd.0001382)

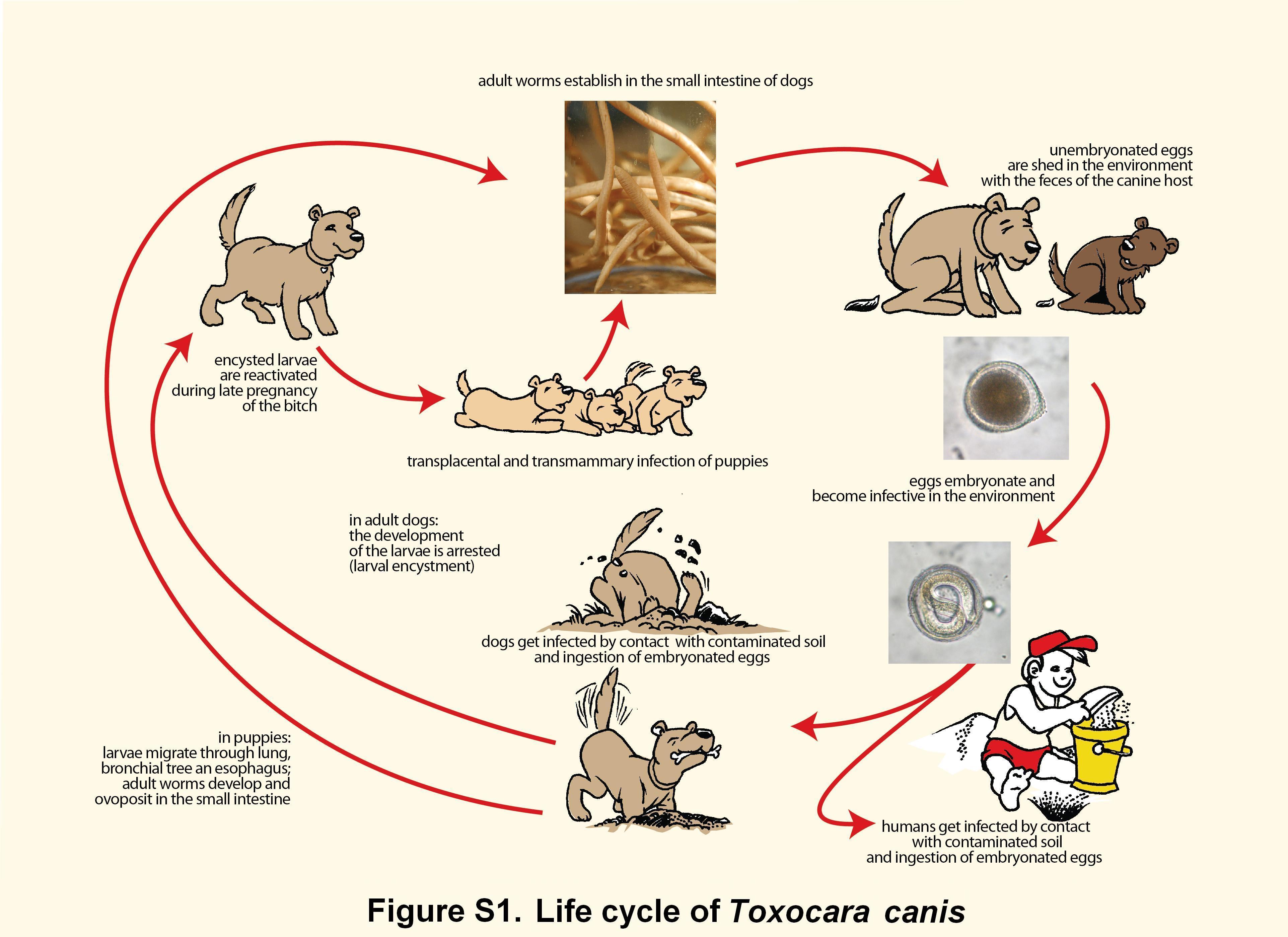

Supplement: Figure S1 — Life cycle of Toxocara canis (JPG) [file pntd.0001382.s001.jpg]
